# Supplementary figures and images for: PolyQ-independent toxicity associated with novel translational products from CAG repeat expansions
Source: PLoS One. 2020 Apr 2;15(4):e0227464. doi: 10.1371/journal.pone.0227464 (PMC7117740; doi:10.1371/journal.pone.0227464)

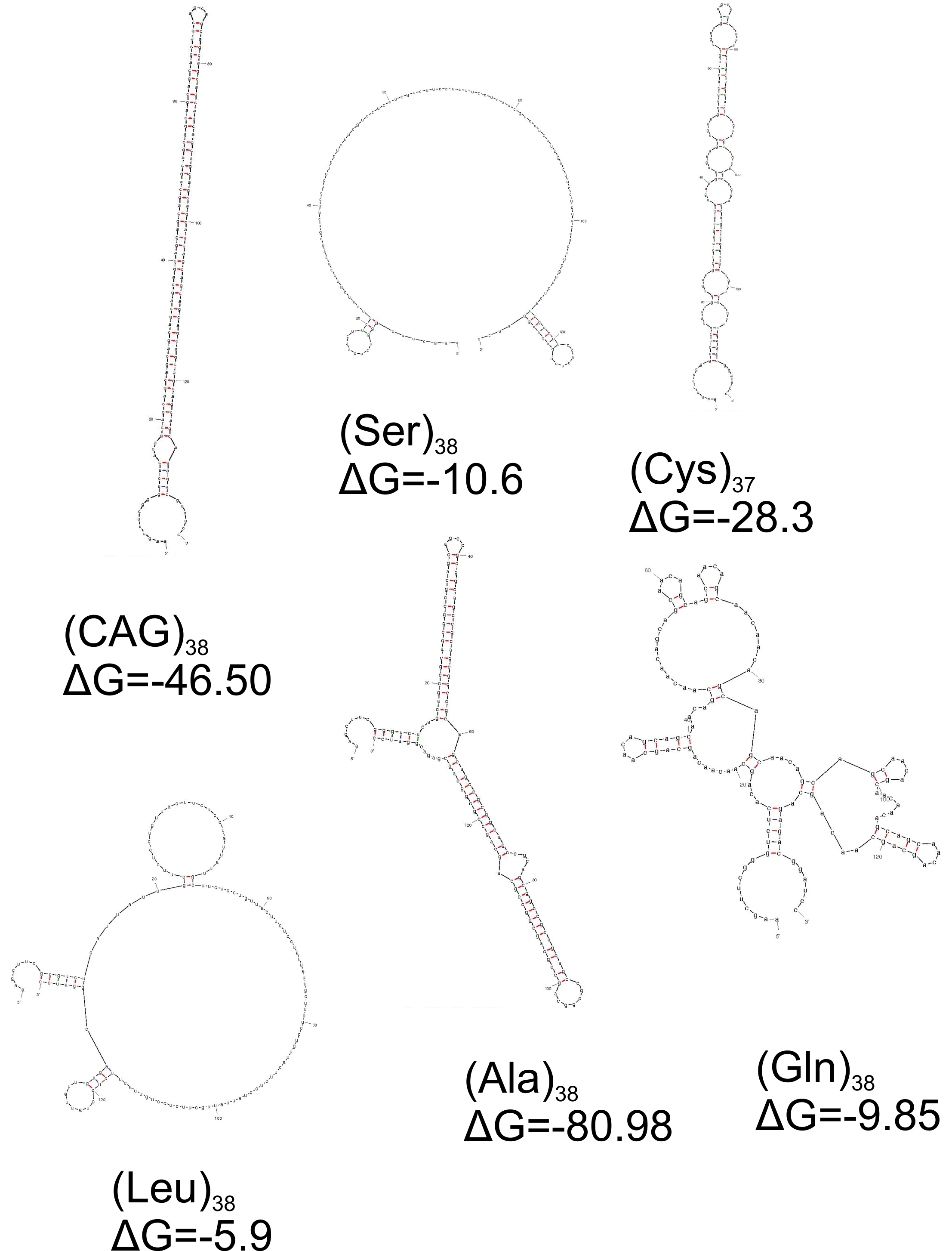

Supplement: S1 Fig — Structure predictions were made with mFold [70]. ‘ΔG’ indicates the free energy difference (kcal) between the folded and unfolded states. Values closer to zero indicate less predicted stable secondary structures. (TIF) [file pone.0227464.s001.tif]

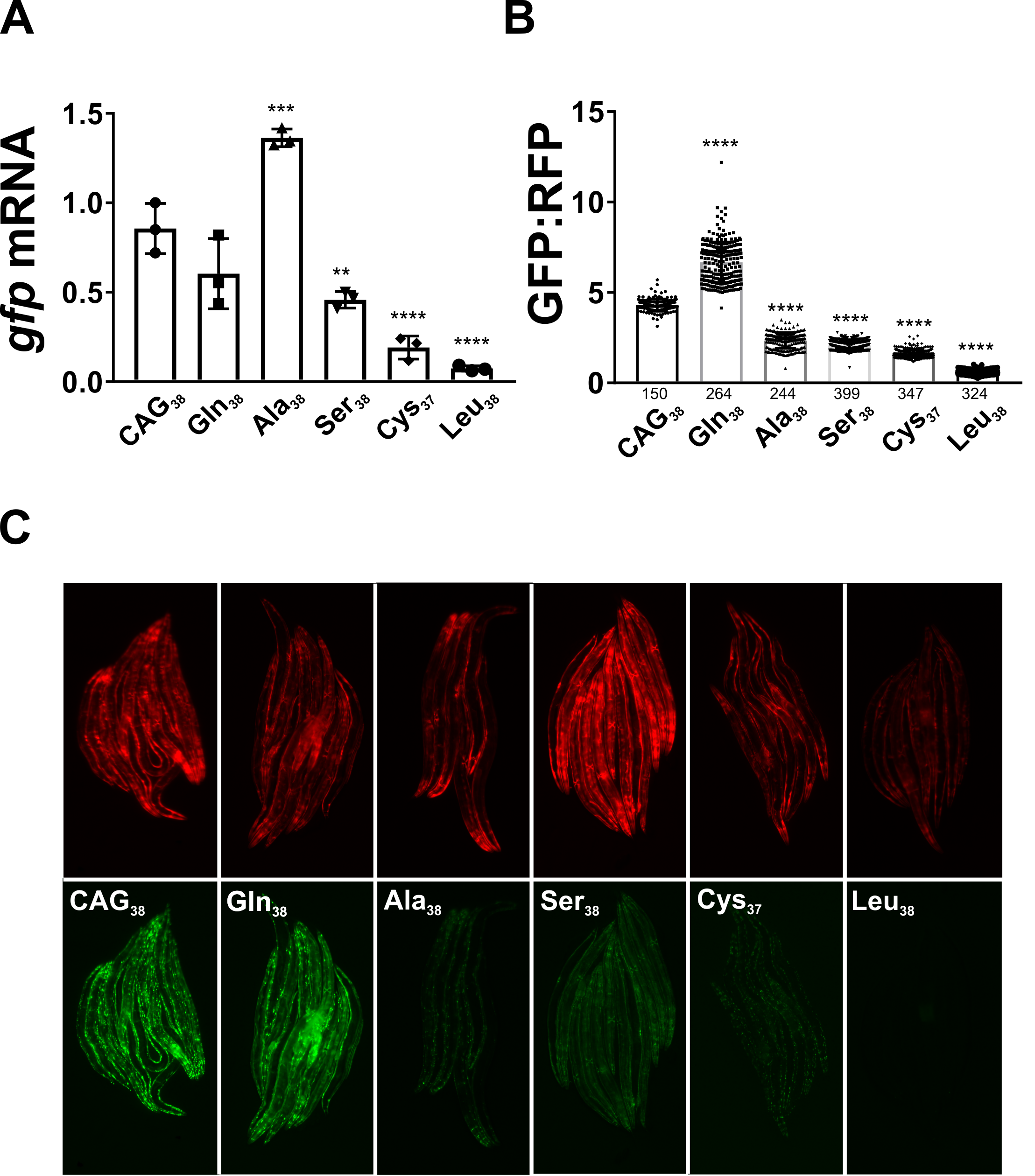

Supplement: S2 Fig — A) qPCR against gfp mRNA for each of the indicated RAN peptide lines expressed under the myo-3 promoter. gfp mRNA levels were normalized levels of the B-actin gene act-2. N = 3 biological replicates, 30–50 worms per genotype per replicate. Data shown are mean ± S.D. normalized to CAG38 expression and individual points reflect values from a single replicate. B) COPAS Biosorter quantification of normalized GFP fluorescence in animals expressing integrated myo-3p::RAN peptide-GFP and myo-3p::mCherry transgenes. RAN peptide-GFP and mCherry are expressed from the same promoter on the same transgene. The GFP signal was normalized against the mCherry signal for each worm. Each point represents the GFP::RFP ratio from a single worm (time of flight ≥400). The number of animals measured is indicated below each bar. **—p<0.01, ***—p<0.001, ****—p<0.0001, One-way ANOVA, Tukey post-hoc multiple comparison test. C) Exposure matched images of day 1 adult animals expressing integrated transgenes with the myo-3p::mCherry and the indicated RAN peptide-GFP. (TIF) [file pone.0227464.s002.tif]
