# Supplementary material for: PolyQ-independent toxicity associated with novel translational products from CAG repeat expansions
Source: PLoS One. 2020 Apr 2;15(4):e0227464. doi: 10.1371/journal.pone.0227464 (PMC7117740; doi:10.1371/journal.pone.0227464)
Supplement: S1 Table — (DOCX) [file pone.0227464.s003.docx]

**Table S1. Nucleotide Sequences of Codon-varied HD RAN polypeptides**

**Red**-HindIII and BamHI cloning sites

| **(Ala)_38_** |
| --- |
| **aagctt**cgggtctcagcagccgcagcggccgcagcagccgcggcagccgcagcggccgcagcagccgcggcagccgcagcggccgcagcagccgcggcagccgcagcggccgcagcagccgcggcagcgagac**ggatcc** |
| **(Cys)_37_** |
| **aagctt**cgggtctcatgttgctgttgctgctgttgctgttgttgttgctgttgctgctgttgctgttgttgttgctgttgctgctgttgctgttgttgttgctgttgctgctgttgctgttgttgtttgagac**ggatcc** |
| **(Ser)_38_** |
| **aagctt**cgggtctcatcttcctcatcgtcctcatcgtcttcctcttcctcatcgtcctcatcgtcttcctcttcctcatcgtcctcatcgtcttcctcttcctcatcgtcctcatcgtcttcctctttgagac**ggatcc** |
| **(Leu)_38_** |
| **aagctt**cgggtctcattattgcttctcctgttacttctcctattattgcttctcctgttacttctcctattattgcttctcctgttacttctcctattattgcttctcctgttacttctcctattattgagac**ggatcc** |
| **(Gln)_38_** |
| **aagctt**cgggtctcacagcaacaacagcagcaacagcagcaacagcaacaacagcagcaacagcagcaacagcaacaacagcagcaacagcagcaacagcaacaacagcagcaacagcagcaacagcagagac**ggatcc** |
| **(Q)_38_** |
| **aagctt**cgggtctcacagcagcagcagcagcagcagcagcagcagcagcagcagcagcagcagcagcagcagcagcagcagcagcagcagcagcagcagcagcagcagcagcagcagcagcagcagcagagac**ggatcc** |
| **(Leu)_11_** |
| **aagctt**cgggtctcattattgcttctcctgttacttctcctattattgagac**ggatcc** |
| **(Leu)_20_** |
| **aagctt**cgggtctcattattgcttctcctgttacttctcctattattgcttctcctgttacttctcctattattgagac**ggatcc** |
| **(Leu)_29_** |
| **aagctt**cgggtctcattattgcttctcctgttacttctcctattattgcttctcctgttacttctcctattattgcttctcctgttacttctcctattattgagac**ggatcc** |
| **(Leu)_90_** |
| **aagctt**ttattgcttctcctactgttattgcttctcctactgttattgcttctcctactgttattgcttctcctactgttattgcttctcctactgttattgcttctcctactgttattgcttctcctactgttattgcttctcctactgttattgcttctcctactgttattgcttctcctactgttattgcttctcctactgttattgcttctcctactgttattgcttctcctactgttattgcttctcctactgttattgcttctcctactgc**ggatcc** |
| **(Cys)_90_** |
| **aagctt**tgttgctgttgctgctgttgttgctgttgctgttgctgttgctgctgttgttgctgttgctgttgctgttgctgctgttgttgctgttgctgttgctgttgctgctgttgttgctgttgctgttgctgttgctgctgttgttgctgttgctgttgctgttgctgctgttgttgctgttgctgttgctgttgctgctgttgttgctgttgctgttgctgttgctgctgttgttgctgttgctgttgctgttgctgctgttgttgctgttgcc**ggatcc** |
| **(Ser)_90_** |
| **aagctt**tcttcctcatcgtcttcctcatcgtcttcctcatcgtcttcctcatcgtcttcctcatcgtcttcctcatcgtcttcctcatcgtcttcctcatcgtcttcctcatcgtcttcctcatcgtcttcctcatcgtcttcctcatcgtcttcctcatcgtcttcctcatcgtcttcctcatcgtcttcctcatcgtcttcctcatcgtcttcctcatcgtcttcctcatcgtcttcctcatcgtcttcctcatcgtcttcctcatcgtcttccc**ggatcc** |
| **(Gln)_90_** |
| **aagctt**caacagcagcaacaacagcaacagcagcaacagcaacaacagcaacagcagcaacagcaacaacagcagcaacaacagcaacagcagcaacagcaacaacagcaacagcagcaacagcaacaacagcagcaacaacagcaacagcagcaacagcaacaacagcaacagcagcaacagcaacaacagcagcaacaacagcaacagcagcaacagcaacaacagcaacagcagcaacagcaacagcaacaacagcaacagcagcaacagcaac**ggatc** |
